# Once- Versus Twice-Daily Measures of Mothers’ Own Milk Biomarkers: Does It Make a Difference in Research and Practice?

**DOI:** 10.1089/bfm.2025.0026

**Published:** 2025-07-07

**Authors:** Marion M. Bendixen, Qinglin Pei, Paula P. Meier, Clarisa Medina-Poeliniz, Rebecca Hoban

**Affiliations:** 1 College of Nursing, University of Florida, Gainesville, Florida, USA.; 2 Department of Pediatrics, Rush University Medical Center, Chicago, Illinois, USA.; 3 Department of Pediatrics, University of Washington, Seattle Children’s Hospital, Seattle, Washington, USA.; 4 College of Nursing, University of Utah, Salt Lake City, Utah, USA.

**Keywords:** human milk, biomarker, secretory activation, breast milk expression, sodium, NICU

## Abstract

**Background:**

Point-of-care (POC) measures of mothers’ own milk (MOM) sodium (Na) concentration are inexpensive, objective measure of secretory activation (SA) achievement with the potential to personalize lactation care, especially among breast pump-dependent mothers with risks for delayed/impaired SA. It is unknown whether POC Na should be measured once or twice daily for research and practice.

**Objectives:**

To determine the need and feasibility of twice- versus once-daily MOM Na measures and examine whether there are differences in MOM Na concentration between morning (AM) versus evening (PM) samples.

**Methods:**

Secondary analysis of a dataset with 312 matched pairs of AM and PM MOM Na measures collected over the first 14 days postpartum in 38 breast pump-dependent mothers of preterm (<33 weeks) infants. Analyses included general linear mixed and regression models, paired rank tests, and descriptive statistics.

**Result:**

Fewer than 50% of subjects had paired samples prior to postpartum day 5 due to MOM being prioritized for infant feedings. Differences between twice-daily measures were significant over postpartum days 1–5, with Na concentrations higher in AM versus PM samples (*p* < 0.0001), a trend that continued (nonsignificant) during postpartum days 6–14. Over postpartum days 1–14, our modeling revealed 80% of the variance in AM measures was predicted by PM measures (*p* < 0.0001).

**Conclusions:**

Feasibility of twice-daily sampling is limited prior to the first 5 postpartum days. Analyses suggest once-daily Na concentrations can be used in research and practice, especially after postpartum day 5. Research priorities include determining the effects of interpump interval and interbreast differences on MOM Na concentrations.

## Introduction

Point-of-care (POC) measurement of mothers’ own milk (MOM) sodium (Na) concentration as a biomarker of secretory activation (SA) achievement holds tremendous potential for research and practice. This is especially true in the neonatal intensive care unit (NICU), where mothers give birth prematurely, are breast pump-dependent, and are often sick themselves with comorbidities that delay and/or impair SA.^1^ In this vulnerable population, lack of timely SA achievement sets the stage for subsequent insufficient MOM volume and lower MOM dose to infants.^2,3^ Thus, the ability to measure SA achievement objectively, accurately, and in real time using ion-selective electrode Na concentrations permits early detection and management of potentially modifiable problems.^4,5^ Previous research has defined SA achievement using MOM Na as either Na ≤16 mM or ≤20 mM, depending upon the specific study.^6–8^ In contrast to this objective measure, maternal perception of *milk coming in* has been shown to be a subjective, inaccurate indicator of SA in breast pump-dependent mothers of NICU infants.^9,10^

In brief, MOM Na concentration is one of four biomarkers of SA achievement (Na, Na:K ratio, lactose, total protein), which reflects the osmotically driven back-and-forth transfer of components between the mammary gland and the maternal circulation during the first postpartum days when paracellular pathways in the mammary gland are patent.^10–12^ Sodium, which passes from the maternal circulation into MOM via patent paracellular pathways, is the highest on postpartum day 1, declines thereafter, and stabilizes with the achievement of SA, rising again only during weaning, MOM stasis, or clinical/subclinical mastitis.^11–13^ Although this downward trend in MOM Na is well-established, the rate of decline is variable among mothers and appears to be affected by factors such as pumping behaviors and maternal comorbidities.^6,10^ Furthermore, two studies of breast pump-dependent mothers of preterm infants revealed a lack of permanence in Na measures of SA achievement through postpartum day 14, indicating a reversal of paracellular pathway closure, thus negatively affecting MOM volume.^6,14^

The within- and between-mother variability in MOM Na concentration raises several logistical questions concerning the implementation of this technology for research and clinical practice. Common among these questions is whether twice-daily versus once-daily MOM sampling is necessary and whether there is any evidence of diurnal (morning versus evening) variability in these measures. The purpose of this study was to examine a unique dataset of twice-daily MOM Na samples that were collected in previously published research.^14–16^ We queried whether there were significant differences between the paired (twice-daily) samples that would support twice- versus once-daily sample collection. We also sought to determine whether there were significant differences between morning (AM) versus evening (PM) samples that would suggest diurnal variability and/or inform optimal timing for sample collection.

## Methods

### Design

This secondary analysis of a previously published study of 39 breast pump-dependent mothers of preterm infants (<33 weeks of gestation at birth) leveraged a unique dataset of paired (AM and PM) MOM biomarkers, including Na concentration, that were prospectively collected from October 2016 to April 2017.^14–16^ The purpose of the original parent study was to determine the feasibility of collecting twice-daily MOM biomarker samples to inform the use of this methodology in a planned randomized conrolled trial (RCT). In this 14-day observational study, mothers collected all pumped MOM in preweighed vials that they labeled with the time and date of pumping and then brought all vials to the NICU. From these pumped MOM containers, the research staff collected 1.8 mL twice-daily (AM and PM) samples, which were frozen until shipment to the University of Western Australia for analysis using ion selective electrodes (Horiba, Japan) to measure Na concentration.^4,14–16^ Per the original study protocol, AM samples were collected from pumpings between 0000 and 1159 hours, and PM samples were collected from pumpings between 1200 and 2359 hours.^14–16^ Samples were not collected if there was insufficient MOM volume or if all available MOM was needed for infant feeding.

The parent study calculated and reported only the mean value of the paired MOM Na concentrations. If two MOM samples were not available, a single once-daily measure was used for analyses. Although the parent study measured multiple MOM biomarkers, Na concentration was most predictive of SA achievement.^10^ Thus, this secondary analysis is limited to paired Na measures collected between postpartum days 1 and 14, a decision that was also informed by the ease of measuring MOM Na using POC techniques.^14–16^

### Sample

In the parent study, 760 MOM Na samples were collected between postpartum days 1–14 and included in the original analysis. Of these, 624 represented part of a paired Na sample, meaning that both a daily AM and PM sample for the same mother were available for analysis. Therefore, 312 paired samples obtained from 38 mothers were included in this analysis. Maternal inclusion criteria have been reported previously.^14,15^ All participants signed consent for the parent study that was approved by the institutional review board at Rush University (Chicago, IL). Subsequently, a data use agreement for analyses conducted in this study was established between Rush University and the University of Florida.

### Procedures and data analysis

The de-identified data were entered into Excel (Microsoft, Redmond, Washington), then cleaned and formatted for statistical analysis. Statistical analysis was performed using Excel and RStudio 2024.04.2 with a significance level of 0.05.^17^ Descriptive statistics were used to summarize the characteristics of the 38 mothers included in this study. Differences between paired Na samples for each postpartum day were compared using Wilcoxon signed-rank tests. To address whether there was a diurnal trend between the AM and PM samples, a mixed-effect model was constructed using PM values to estimate AM values for Na concentration, with subject and postpartum days as random effects. This analysis was conducted for the entire 1–14 postpartum day period.

## Results

Characteristics of the original sample of 39 mothers have been previously published.^14–16^ For this secondary analysis, maternal/infant characteristics for the 38 mothers with paired MOM samples are summarized in Table 1. Of note, the sample was primarily minority (Black = 55%; Hispanic = 21%; White = 19%), low-income (68%), and overweight or obese (68%), with 79% of the sample having at least one comorbidity, including chorioamnionitis, diabetes, postpartum hemorrhage, obesity/overweight, or preeclampsia.

**Table 1.** Maternal/Infant Characteristics (*n* = 38)

Characteristic	Mean [standard deviation] or *n* (%)
Infant gestational age (weeks)	28.8 [2.7]
Infant birthweight (g)	1263 [491]
Infant male sex	25 (66%)
Maternal age (years)	28.6 [6.1]
Maternal race/ethnicity	
Black, non-Hispanic	21 (55%)
White, non-Hispanic	7 (18%)
White, Hispanic	8 (21%)
Other Asian/American Indian/Alaskan Native	2 (5%)
WIC eligible^a^	26 (68%)
Employed prior to delivery	24 (63%)
BMI^b^ (kg/m^2^) (prepregnancy)	28.6 [9.0]
BMI category	
Normal weight (BMI < 25)	10 (26%)
Overweight (BMI 25–< 30)	7 (18%)
Obese (BMI ≥ 30)	19 (50%)
Received antenatal steroids	36 (95%)
Bedrest during pregnancy	28 (74%)
Preterm labor	32 (84%)
Cesarean delivery	15 (40%)
Primigravida	9 (23%)
Number of comorbidities^c^	
0	8 (21%)
1	17 (45%)
2	11 (29%)
3	2 (5%)

a WIC: Special Supplemental Nutrition Program for Women, Infants, and Children (socioeconomic status proxy).

b BMI: body mass index.

c History of chorioamnionitis, diabetes, postpartum hemorrhage, obesity/overweight, and/or preeclampsia.

Characteristics of the paired Na samples from 38 mothers over postpartum days 1–14 are summarized in Table 2. Prior to postpartum day 5, fewer than half of the mothers had twice-daily Na samples available for analysis due to lack of MOM or prioritization of MOM for infant feeding. Daily mean absolute differences between AM and PM Na concentrations for postpartum days 1–5 are significantly larger than for subsequent days (Table 2). Paired rank tests (Table 2) were conducted between aggregate AM and PM values for each of the 14 postpartum days, revealing statistically significant differences over postpartum days 2–7 with no differences for postpartum days 8–14.

**Table 2.** Paired Sample Characteristics by Day (312 Samples from 38 Mothers)

Postpartum day	Number paired samples	AM Na concentration (mM)				PM Na concentration (mM)				Mean absolute AM/PM difference	Paired rank test
		Mean	Median	Min	Max	Mean	Median	Min	Max		*p*
1	1	50.00	50.00	50.00	50.00	56.52	56.52	56.52	56.52	6.52	N/A
2	7	50.59	52.17	34.35	69.57	33.73	33.91	14.57	63.04	16.86	0.023
3	15	24.75	18.70	11.52	91.30	18.58	14.57	6.96	65.22	6.61	0.004
4	19	18.60	16.09	7.39	60.87	15.78	12.83	6.52	47.83	3.26	0.004
5	31	18.75	15.43	6.52	58.70	16.06	13.26	6.30	41.74	3.48	0.002
6	29	14.93	12.83	6.52	45.22	13.19	12.39	6.09	31.74	2.86	0.012
7	24	14.37	12.72	5.65	54.35	12.25	10.22	6.09	38.26	2.90	0.01
8	27	15.47	11.08	6.96	37.17	14.44	10.65	6.74	35.65	2.99	0.12
9	27	14.55	12.17	5.87	31.74	14.82	13.26	6.09	34.35	2.74	0.69
10	27	14.89	11.96	4.35	58.70	14.11	10.87	4.78	52.17	2.93	0.28
11	29	13.69	11.52	5.43	43.04	11.58	11.09	5.22	21.96	3.13	0.10
12	29	11.28	9.78	5.87	23.91	12.38	10.00	5.22	30.65	2.41	0.23
13	26	14.72	11.63	4.78	44.22	14.19	11.52	5.65	45.65	3.75	0.15
14	21	13.31	12.17	5.65	24.78	12.85	11.09	5.65	24.78	3.34	0.51

AM, morning; Max, maximum; Min, minimum; Na, sodium; N/A, not applicable; PM, evening.

**FIG. 1.**
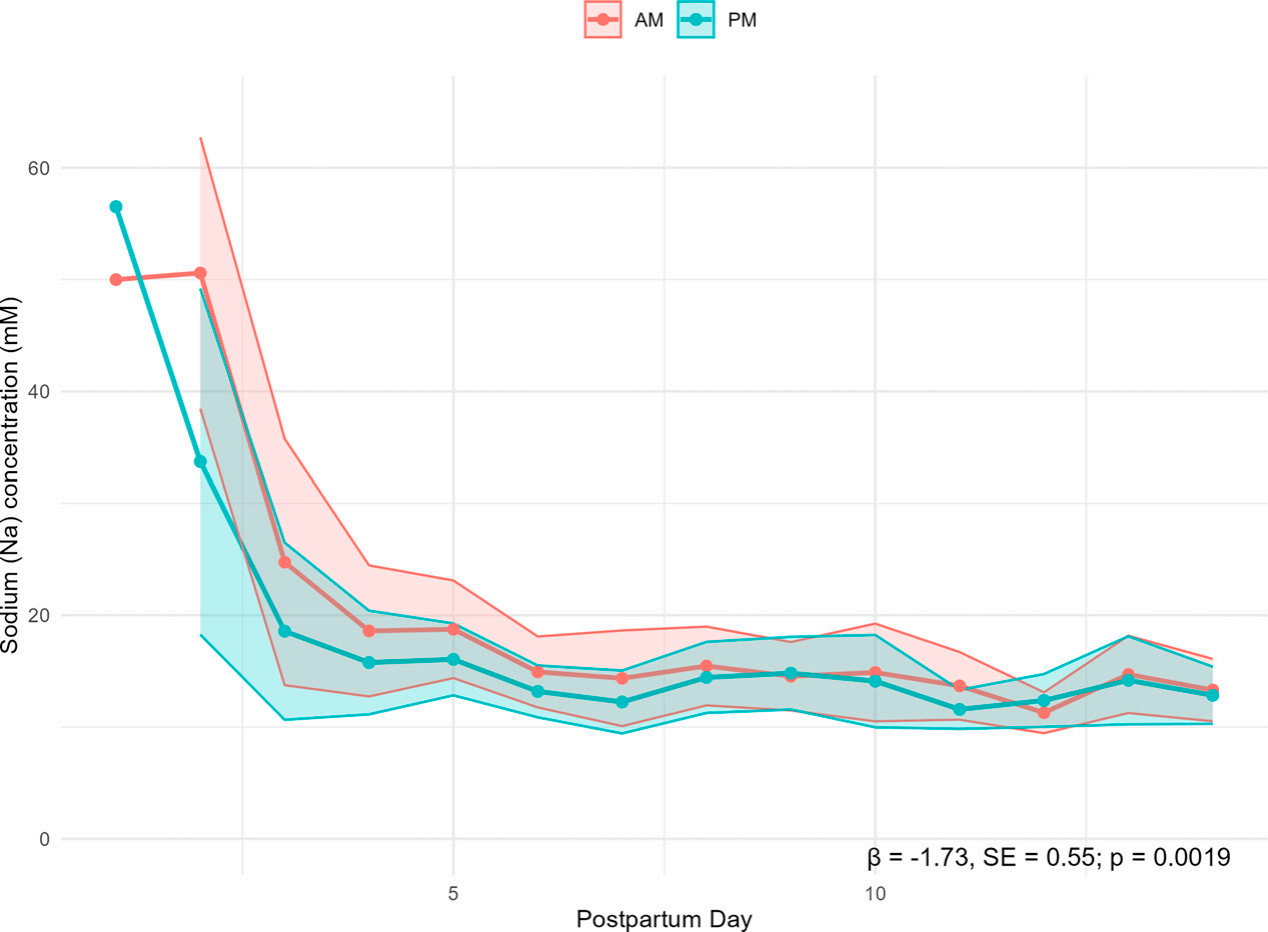
Comparison of AM versus PM sodium (Na) mean concentrations over postpartum days 1–14. AM, morning; PM, evening; Shaded areas represent 95% confidence intervals.

**FIG. 2.**
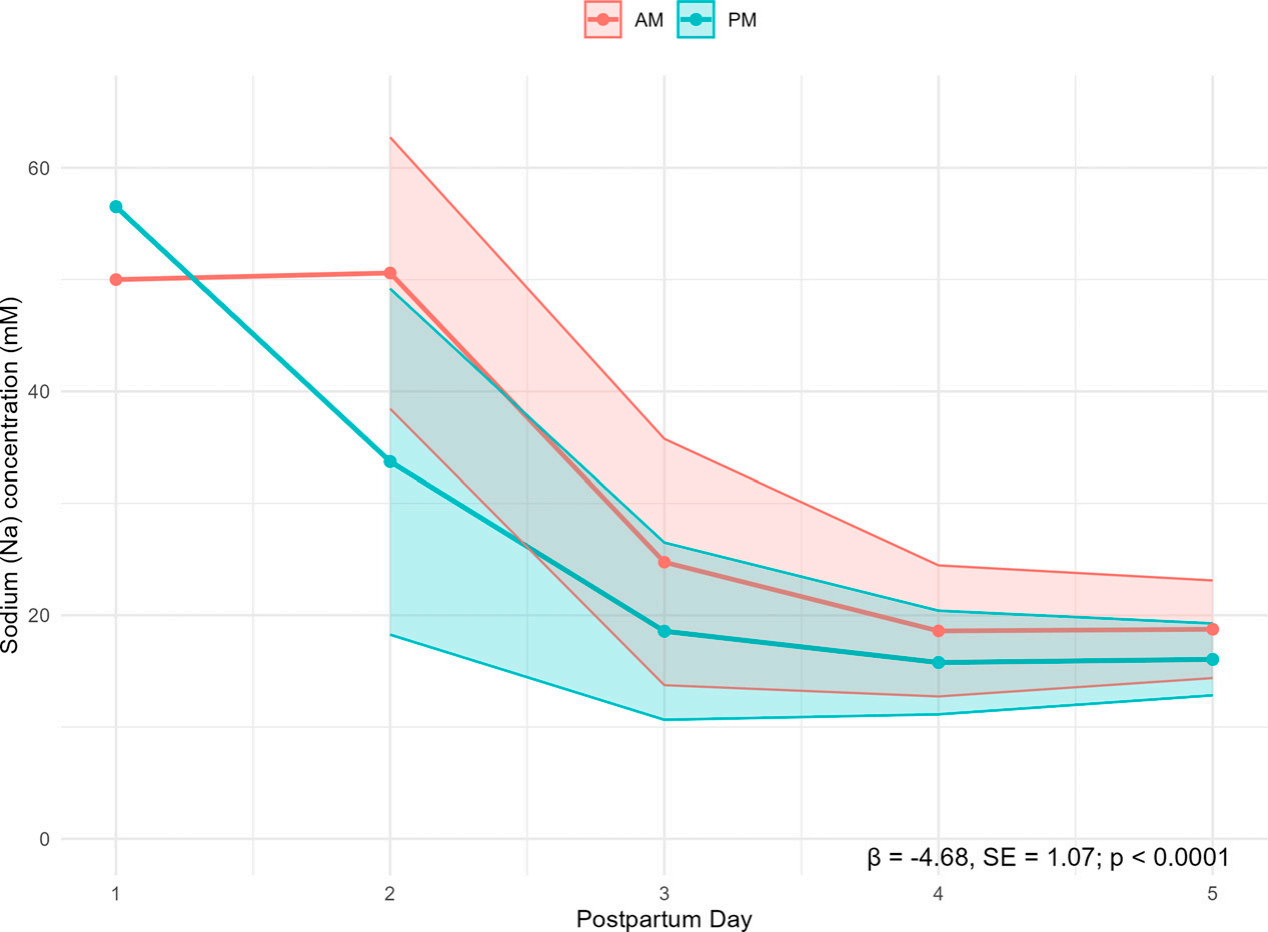
Comparison of AM versus PM sodium (Na) mean concentrations over postpartum days 1–5. Shaded areas represet 95% confidence intervals.

**FIG. 3.**
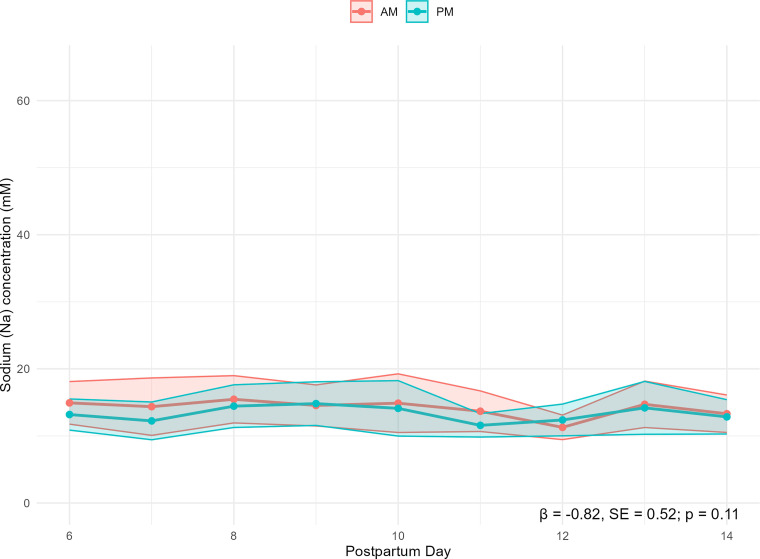
Comparison of AM versus PM sodium (Na) mean concentrations over postpartum days 6–14. Shaded areas represent 95% confidence intervals.

Our mixed-effects model examined the relationship between Na concentration and time of day and revealed a statistically significant difference (*p* = 0.002) between AM and PM Na concentration over postpartum days 1–14 (Fig. 1). This difference was primarily driven by a significant effect within the first 5 postpartum days (*p* < 0.0001, Fig. 2), with no significant difference observed for days 6–14 (*p* = 0.11, Fig. 3).

To determine whether there was a diurnal variation in MOM Na concentrations, we constructed a mixed-effect regression model to predict AM values using PM values. The adjusted *R*^2^ for postpartum days 1–14 was 0.80, indicating that 80% of the variability in AM Na concentration was predicted by PM Na concentration (*p* < 0.0001).

## Discussion

To our knowledge, this is the first study to report differences between AM versus PM MOM Na concentrations in pairs of MOM samples collected from the same mother over the first 14 postpartum days. In this study of 312 paired AM and PM MOM Na samples obtained from 38 breast pump-dependent mothers of preterm infants, we found statistically significant differences between AM and PM Na concentrations over postpartum days 1–14. However, these differences were confined to postpartum days 1–5, with no differences observed for postpartum days 6–14. Our mean absolute differences and paired rank tests reveal similar findings with respect to greater variability between AM and PM samples during the first postpartum week than the second. However, our regression model revealed that PM Na concentrations predicted 80% of the variance in AM Na concentrations, suggesting that in most instances, once-daily values can suffice for research and practice.

### Variability in MOM Na over time

Figure 1 and Table 2 reveal that both AM and PM Na concentrations follow time-dependent trends described in the classical literature, in which the Na concentration is highest in the first postpartum day(s), then declines and stabilizes by the end of the first postpartum week.^13,18^ This pattern reflects the gradual closure of the paracellular pathway in the mammary gland such that Na from the maternal circulation cannot cross readily into MOM.^12,13,19^ We found that both AM and PM measures in this population of pump-dependent mothers of preterm infants trended toward SA achievement between postpartum days 5 and 6. Only half of the cohort had sufficient MOM volume prior to postpartum day 5 to allow for twice-daily sample collection, suggesting very low MOM volumes and therefore lack of SA achievement for the remaining 19 mothers (Table 2 for *n* by postpartum day). Our data are consistent with those of Hoban et al.^6^ who reported a median postpartum day of 5.8 for SA achievement (Na <16 mM) in a comparable sample of 29 mothers of preterm infants, with no mother achieving SA prior to postpartum day 4. These findings inform future studies with respect to potential for obtaining twice-daily samples during the first postpartum week. Specifically, Na concentration changes most rapidly during this time, and twice-daily samples may detect SA achievement earlier than once-daily samples. Although this difference may be important for studies comparing the timing of SA achievement, twice-daily sample collection may not be feasible due to insufficient MOM volume.

### AM versus PM MOM Na concentrations

Table 2 reveals a statistically significant difference between AM and PM Na samples between postpartum days 1 and 7, with no significant differences thereafter. Figures 1–3 show a similar pattern, in that significant differences over the 14-day study period are confined to the first 5 postpartum days. Nonetheless, the statistically significant prediction model (*R*^2^ = 0.8, *p* < 0.0001) indicates that PM measures predict AM measures over the first 14 postpartum days. While it is tempting to assume an underlying diurnal mechanism to explain differences between AM and PM Na concentrations, there is an absence of biological support for this hypothesis. We speculate that breast pump dependency and our sampling protocol may have influenced these differences, as detailed below.

Unlike breastfeeding a healthy term infant who stimulates the mammary gland and removes MOM on the basis of hunger, breast pump dependency provides the mother with flexibility as to when to pump. During the early postpartum period, these mothers are often sick and may be overwhelmed with the stress surrounding their infant’s NICU admission. Thus, they tend to pump less frequently and regularly than recommended in all studies to date.^6,20–22^ We have previously reported the positive association between the frequency of breast pump use and the achievement of SA, defined as Na <16 mM.^6^ However, no previous study has examined the effect of interpump interval (e.g., the time between the end of one pumping session and the end of the next) on MOM Na concentration, and interpump interval was not controlled for in the original parent study for this analysis.

Longer interpump intervals can result in MOM stasis, reopening paracellular pathways and permitting maternal serum Na to pass into MOM.^19^ Thus, our differences between AM and PM Na concentrations may be due to pumping regimens that prioritize frequent pumping during daytime hours and longer stretches of nighttime sleep for these vulnerable mothers. As such, first AM pumpings would likely be higher in Na due to lack of overnight MOM removal/mammary gland stimulation, whereas PM samples would reflect more complete breast emptying as a result of more frequent pumping. Understanding the effect of interpump interval on MOM Na concentrations is a research priority to inform the timing of Na sample collection within a regime of breast pump dependency. Of note, other MOM components, particularly lipids that were once thought to be diurnal, have been linked to interpump or interfeed intervals.^23^

Another potential factor influencing AM and PM Na concentration differences is that samples were collected within a 12-hour range, rather than at 12-hour intervals, so theoretically, some samples could reflect pumpings separated by only a few hours. While it would be desirable to collect 12-hourly samples, the feasibility of doing so with this population would be challenging due to irregular pumping times and concern for subject burden. Additionally, the mean postpartum day for this sample to achieve Na <16mM was 5.5, with 5 (14%) mothers first achieving SA on postpartum days 11 or 12. Describing these data in the aggregate by postpartum day, as we have done in Table 2 and Figures 1–3, reflects the inclusion of the wide range of samples from mothers who had already achieved SA, those who had not yet achieved it, and those who initially had very low volumes and who might be providing a first MOM sample on that day. Thus, the high variability between both AM and PM Na concentrations among individual mothers must be considered when applying findings to the clinical setting and/or informing effect size for subsequent studies.

### Research gaps and priorities

These findings highlight research gaps and priorities for the use of POC Na concentrations in both research and practice. Although a strength of this study was the large number of paired samples from 38 breast pump-dependent mothers of preterm infants, the proportion of missing samples presumably due to MOM unavailability from days 1 (96%), 2 (70%), 3 (53%), and 4 (50%) emphasizes the difficulty in obtaining twice-daily measurement in this population. A research priority is to understand the potential impact of interpump intervals on MOM Na concentration, which is needed to inform appropriate sample collection, as well as clinical recommendations for pumping. Furthermore, the parent study for our analysis collected MOM samples from the two breasts combined. It is feasible that interbreast differences in MOM Na concentrations are masked by a composite Na measure from the two breasts combined, especially as the gland is transitioning from SA (hormonal control) into the autocrine/paracrine control of lactation. Identification of interbreast differences is an actionable clinical priority that might inform the effectiveness of breast pump use, such as breast shield placement and sizing. A final research gap is that our study examined only Na concentrations due to their POC measurement utility. Our findings cannot be generalized to other MOM biomarkers that represent the upregulation of transcellular pathways in the mammary gland, including lactose, citrate, total protein, and fat.

## Conclusions

We conclude that twice-daily MOM Na concentration may be beneficial in targeting an exact time for the achievement of SA, especially for comparisons that involve lactation research interventions. However, the lack of MOM available for testing during the early postpartum days in the majority of mothers suggests limited feasibility. Our data inform several priorities and research gaps for the use of Na POC measures in breast pump-dependent mothers of preterm infants. Topping the list is testing the impact of interpump intervals on MOM Na concentration to determine whether this measure should be standardized in clinical and research protocols. Also important for future research is testing MOM Na from the two breasts separately, as findings may inform clinically actionable remedies in a timely manner. Finally, our analysis suggests PM Na concentration values explain 80% of the variance in AM values. Therefore, although twice-daily testing would be ideal if MOM volumes were adequate, our data indicate that in most instances, once- versus twice-daily measures are acceptable strategy.
